# Completeness of Reporting and Intervention Description in Articles on Psychological Interventions for Pediatric Patients with Adolescent Idiopathic Scoliosis: A Meta-Research Study

**DOI:** 10.3390/healthcare13222872

**Published:** 2025-11-12

**Authors:** Petar Kaliterna, Marija Franka Žuljević, Ana Marušić, Ivan Buljan

**Affiliations:** 1 Department of Physical Medicine and Rehabilitation, Polyclinic for the Rehabilitation of Persons with Developmental Disabilities, 21000 Split, Croatia; 2 Centre for Evidence-based Medicine, School of Medicine, University of Split, 21000 Split, Croatia; marija.franka@gmail.com (M.F.Ž.); ana.marusic@mefst.hr (A.M.); 3 Department of Psychology, Faculty of Humanities and Social Sciences, University of Split, 21000 Split, Croatia; ibuljan@ffst.hr

**Keywords:** adolescent idiopathic scoliosis, psychological interventions, reporting guidelines, reproducibility, research quality

## Abstract

**Highlights:**

**What are the main findings?**

- Reporting of psychological interventions for scoliosis is frequently incomplete.
- Most studies failed to fully describe critical intervention elements.
- General reporting quality was poor in psychological intervention randomized trials.
- Reporting quality in observational studies was inconsistent or insufficient.

**What is the implication of main findings?**

- Standardized guidelines are needed for transparency in the research field of scoliosis.

**Abstract:**

**Introduction**: Adolescent idiopathic scoliosis (AIS) presents not only physical but also psychological challenges for affected patients, frequently requiring comprehensive management that includes psychological interventions. Accurate and transparent reporting of interventions is essential to support reproducibility, facilitate clinical translation, and advance research quality. However, the completeness of intervention reporting and adherence to standardized guidelines in published studies on psychological interventions for pediatric AIS patients remains unclear. **Methods**: We searched Medline, PsycINFO, and Cochrane Central Register of Controlled Trials (CENTRAL) for studies involving psychological interventions in AIS. Intervention reporting was analyzed using the Template for Intervention Description and Replication (TIDieR) checklist. Adherence to reporting guidelines was assessed for different study designs. **Results**: We identified 18 studies, which had suboptimal reporting of interventions. For key TIDieR items, all studies reported the brief name and rationale, but completeness for other elements varied: methods (14/18 studies), materials (10/18), provider (6/18), and mode of delivery (8/18) were described inconsistently. Setting was reported in only 3/18 studies, whereas the details about tailoring, modifications, and fidelity were largely lacking or deemed non-applicable. For eight randomized trials, several critical CONSORT items, such as trial design, randomization procedures, blinding, and trial registration were often unreported. Among five observational studies, reporting of STROBE key elements such as study design, setting, eligibility criteria, and funding was more consistent, but methods addressing bias, participant flow, missing data, and category boundaries for variables were largely insufficient. Similar gaps were observed for relevant TREND checklist items for nonrandomized intervention studies. **Conclusions**: The reporting of psychological interventions for AIS in the literature is frequently incomplete, especially for intervention details essential for the reproducibility of the intervention and assessment of risk of bias. Adoption of standardized reporting guidelines is necessary to improve intervention transparency, replicability, and translation into clinical practice. Future research should focus on prospective evaluations of reporting guideline implementation and its impact on research quality in this field.

## 1. Introduction

Adolescent idiopathic scoliosis (AIS) [1] is a disorder that can result in substantial aesthetic difficulties and mental discomfort for affected adolescents, including anxiety and depression, even though the majority of young people with scoliosis do not have clinical symptoms [2,3]. Surgery, braces, and observation are all possible forms of treatment [1], but the recent literature has examined the effectiveness and importance of psychological interventions due to the fact that, despite limited high-quality research, current evidence highlights the critical role of multidisciplinary approaches that include psychological care to address the complex biopsychosocial needs of young people with scoliosis [4].

In physical and rehabilitation medicine, where many treatments are non-pharmacological, clear and comprehensive reporting of interventions is critical. The cornerstone of contemporary evidence-based medicine is the thorough and transparent presentation of methodological procedures, including the study intervention, to ensure the study’s reproducibility in research and in practice, and to assess a clinical trial’s validity [5]. Inadequate descriptions can impair both implementation and evaluation, ultimately limiting patient benefit [6,7,8].

The description of both psychological and non-psychological interventions in journals is of notably low quality [6,9,10], especially in the field of physical and rehabilitation medicine [11,12]. This is particularly problematic in scoliosis research, where no prior studies have specifically assessed the completeness of psychological intervention reporting. A systematic review from 2023 by van Niekerk et al., which included 13 articles of psychosocial interventions for patients with AIS, concluded that it was difficult to draw conclusions about the efficacy of the interventions because of shortcomings in the reporting of studies [13].

Despite the existence of methodological guidelines, such as the EQUATOR Network’s initiatives to enhance the quality and transparency of health research, we can see that reporting quality remains inconsistent [14,15]. One widely used guideline, the 2014 Template for Intervention Description and Replication (TIDieR) checklist, was developed to improve the reporting of non-pharmacological trials [16,17]. However, TIDieR primarily addresses physical and behavioral interventions and may not sufficiently capture the complexity of psychological components. This highlights a methodological gap, as psychological interventions, particularly in the context of scoliosis treatment, often lack specific guidance to ensure accurate and comprehensive reporting.

The aim of this study was to assess the quality of reporting of psychological interventions in patients with AIS. We used the TIDieR checklist for the quality of intervention description, and EQUATOR Network’s guidelines, depending on the study type, to assess the overall quality of reporting within the included study designs.

## 2. Materials and Methods

### 2.1. Study Design

This was a meta-research study. Our data sources were publicly available published articles that involve psychological interventions in patients with adolescent idiopathic scoliosis.

### 2.2. Identification of Studies

The search strategy outlined by van Niekerk et al., who published a comprehensive overview on the topic of pediatric scoliosis [13], was used to identify studies. The search strategy included a combination of the words “psychosocial intervention”, “pediatric”, and “scoliosis” using standardized subject and free-text terms, including synonyms and alternative spellings. All databases available to us were searched, including the following: Medline, PsycINFO, and Cochrane Central Register of Controlled Trials (CENTRAL). We included the 13 articles previously identified in van Niekerk’s study, which covered the period from database inception to March 2022. Building on this, we conducted an updated comprehensive search from March 2022 to October 2024 to capture any additional relevant studies. During this process, two systematic reviews published in 2023 were also identified [4,18], and the studies included in these reviews were hand-searched and assessed for eligibility.

### 2.3. Participants

The inclusion criteria for our study were all studies where patients with adolescent idiopathic scoliosis received a psychological intervention. All types of interventional studies were included: randomized and non-randomized, double-blinded, blinded or non-blinded. We excluded studies that did not involve psychological intervention, studies that were found in non-peer-reviewed articles, and studies that did not include an intervention.

### 2.4. Variables

We assessed the completeness of the TIDieR checklist, where each of the 12 TIDieR items was rated as “yes” if they were reported in full, “no” if they were not reported at all, and “unclear” if they were reported with insufficient precision and details. If the item was not planned to be included in the intervention based on what the authors specified in their manuscript, we rated it as “non-applicable” (N/A).

We also assessed the reporting completeness of each study based on the CONSORT from 2010, STROBE or TREND checklists [19,20,21], depending on the study type. Items from EQUATOR guidelines checklists were rated with “yes”, “no” and “partial” if some of the requested data from respective Item was missing, but other requirements were matched.

We also collected the information on the type of study, the name and impact factor of the journal where it was published and the year of publication of the study.

### 2.5. Data Sources

The identification of corresponding publications, a pilot extraction and assessment of three studies for all variables was independently performed by two of the authors (PK and MFŽ). After comparing their results, a clear protocol was established to minimize subjective interpretation during data extraction and analysis. Once evaluation criteria for each item were agreed upon (Supplementary Material S1), both authors independently assessed each guideline. There were no discrepancies between their judgments (Cohen’s κ = 1.0), indicating perfect inter-rater agreement. A third author (IB) reviewed and confirmed all results, and no further resolution was necessary.

### 2.6. Statistical Methods

All statistical analyses were performed using JASP software v. 0.13.1.0 (JASP Team, 2023, Amsterdam, the Netherlands) and R statistical program (R Core Team version 4.1.1, 2021). For numerical variables, the Shapiro–Wilk test was used to assess the normality of data distribution, which determined which measure of central tendency (mean or median with corresponding 95% confidence intervals) was used to express continuous variables. The level of significance cut-off for all statistical tests was set to *p* < 0.05.

## 3. Results

**Figure 1:**
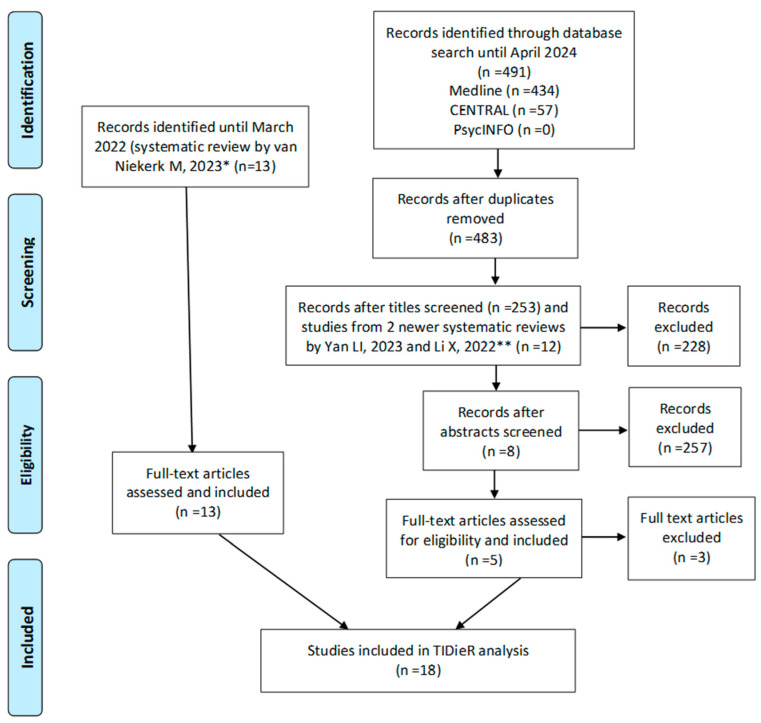
Flow chart of study selection, * [13], ** [4,18].

A total of 491 articles were identified through the search strategy (Figure 1). After removing duplicates, 483 titles were screened, resulting in a total of 253 articles and 2 newer systematic reviews [4,18], from which we also included 12 articles in the analysis. Furthermore, abstracts of the remaining articles were screened, which yielded eight articles for full-text review, from which five were selected [22,23,24,25,26] that met our selection criteria. In addition, 13 articles [27,28,29,30,31,32,33,34,35,36,37,38,39] from the systematic review by van Niekerk et al. [13] were also included. This left a total of 18 articles for data extraction.

Thirteen of the eighteen articles that we included were controlled trials, of which eight were randomized (RCT) [24,25,33,34,35,36,37,39], and five were non-randomized studies of interventions [27,29,30,31,32]. Four articles were cohort studies [22,23,26,38], and one article was a cross-sectional study [28]. Publication dates ranged from 1985 to 2023, with only one publication from 1985, before the first reporting guidelines were published (CONSORT in 1996) [40], while the rest were published from 2003 onwards. Journal impact factor in the year of publication ranged from 0.2 to 4.8, with two papers published in journals that did not have an impact factor at that time.

### 3.1. Completeness of Intervention Descriptions in the Published Articles

The best reported parts of the articles were the name of the study and the rationale for undertaking the study (Items 1 and 2), which were reported in all 18 articles. The authors mostly mentioned materials and methods used in the intervention (Items 3 and 4); however, in six studies, the materials were not explained in detail, or it was not stated where they could be accessed. Also, the methods were unclearly explained in three studies. Only a few studies did not list their materials or methods at all.

Items 5, 6 and 7, which refer to who provided intervention and how and where the intervention was conducted, were the least reported, with only three papers clearly reporting where the intervention was carried out and ten papers that did not mention the place of the intervention at all. The description of who provided the intervention and how was somewhat better, although less than half of the studies (six for who provided and eight for how) fully described these parts of the intervention. The timing and frequency of the intervention (Item 8) were predominantly recorded; however, five publications lacked critical details in several aspects of the intervention. The study from 1985 did not adequately report any of the mentioned items.

TIDieR items 9–12, which refer to the personalization and modification of the intervention and the assessment of adherence in most studies, were not planned to be included in the intervention as described by the study authors, so we could not apply our analysis to them. The full data on all of the items and their reporting across the included studies can be found in Table 1, with full reasoning and explanation (see Supplementary Material S2).

### 3.2. Overall Reporting Completeness According to Relevant EQUATOR Guidelines

To maintain clarity and focus, we chose to report in tables only those EQUATOR guidelines checklist items that were not adequately reported. Other items not mentioned in Table 2, Table 3 and Table 4 were mostly adequately reported, with full results and reasoning available in Supplementary Material S3.

#### 3.2.1. Adherence to CONSORT Checklist

A total of eight randomized controlled trials from 2003 to 2018 were evaluated for adherence to CONSORT reporting items. The completeness of reporting varied widely across the assessed items (Table 2).

Comparatively low reporting rates were noted for identification as a randomized trial in the title, sample size determination, recruitment and follow-up dates, and the number of participants included in each analysis, with each item reported in only three studies. The mechanism used to implement the random allocation sequence and generalizability of trial findings were described in two studies. Several critical methodological items were infrequently reported, as only a single study described the trial design (including allocation ratio), specified the type of randomization and any restrictions, reported who generated the random allocation sequence, who enrolled participants, who assigned interventions, and detailed who was blinded after assignment to interventions and how blinding was achieved, respectively. Similarly, the registration number and name of trial registry, as well as information on where the full trial protocol could be accessed, were each reported only in a single study. This was concerning considering that trial registration has been mandatory since 2005 [41], and most of the analyzed studies (5/8) were published after this time point.

#### 3.2.2. Adherence to STROBE Checklist

A total of five studies were assessed for adherence to items from the STROBE checklist: four for STROBE for cohort studies and one for STROBE for cross-sectional studies. The proportion of studies adequately reporting each STROBE item varied considerably (Table 3).

Key elements such as study design, setting, eligibility criteria, generalizability, and funding were adequately reported in three out of a total of five studies. Efforts to address bias, loss to follow-up, reasons for non-participation, and reporting of estimates were present in two studies. However, reporting was poor for several critical items: only one study described data sources, study size calculation, participant flow, or numbers at each stage. Notably, none of the included observational studies explained how missing data were addressed, indicated missing data per variable, nor reported category boundaries for categorized variables.

#### 3.2.3. Adherence to TREND Checklist

Assessment of reporting quality across the TREND checklist items revealed considerable variability. For the participant recruitment and setting, participant flow, and baseline data items, none of the five studies provided complete information. However, all studies offered partial reporting, missing either the mention of the location and sampling strategy, or information on the periods of recruitment. Similarly, no studies fully reported on sample size or blinding, which were not mentioned in any of the five studies. The dates defining periods of recruitment and follow-up were more consistently addressed, with three out of five studies providing complete information (Table 4).

## 4. Discussion

Our analysis revealed that the overall reporting quality of psychological intervention studies for patients with scoliosis was largely inadequate. This applied both to general reporting standards outlined in the EQUATOR Network guidelines and the completeness of intervention descriptions assessed using the TIDieR checklist. Critical methodological details, such as the materials and procedures used, as well as how, where, and by whom interventions were delivered, were frequently missing or insufficiently described, limiting reproducibility and clinical applicability. For example, fewer than half of the studies clearly identified the provider or setting of the intervention, and only three fully addressed the location of delivery. However, to provide a more balanced interpretation, it is important to acknowledge that, while reporting among observational studies varied, a majority did include core elements such as study design, setting, and funding, with key gaps primarily observed in the transparency of methodological details.

Our study was limited by an overall small number of included studies, primarily due to limited research on psychological interventions for adolescent idiopathic scoliosis. Additionally, we included one study published in 1985 because it met all the predefined inclusion criteria despite predating the first reporting guidelines established in 1996, while all other studies were published from 2003 onwards. Since reporting standards and methodological rigor have evolved over time, the 1985 study may differ in quality and transparency. Also, due to lack of institutional access, we could not repeat searches for the Embase and EBSCO Cumulated Index to Nursing and Allied Health Literature (CINAHL) databases from the search strategy by van Niekerk et al. This may have limited the comprehensiveness of our literature search and introduced a degree of selection bias. However, we mitigated this by conducting extensive searches in other major and most relevant databases such as MEDLINE and carefully screening the reference lists of included studies, making our study search highly likely to be representative. Additionally, we decided to also include any relevant studies from other identified systematic reviews to ensure full representation of all relevant data and further increase the representativeness of the findings. We do not claim to make any final conclusions but rather provide guidance for better reporting in future clinical studies in this area. Also, we did not use new and more specific guidelines for our analysis like the TIDieR-Rehab checklist and extensions of CONSORT, which can add value for further research.

Many authors have emphasized the importance and numerous benefits of using the TIDieR checklist to improve the understanding, reproducibility of research and adequate translation into practice in physical and rehabilitation medicine [42,43]. Some studies in physical and rehabilitation medicine, as well as orthopedic surgery, found that interventions are underreported in clinical trials according to TIDieR, jeopardizing the external validity of trials and making it difficult for clinicians and researchers to replicate them [44,45]. On the other hand, one study in the field of physical and rehabilitation medicine concluded, using the TIDieR checklist, that interventions were moderately described in trials and provided enough information to guide the decision making [46]. To our knowledge, this is the first study to provide an overview of intervention descriptions in psychological interventions in patients with scoliosis. Therefore, our findings are generally comparable to those of previous studies in various fields of medicine [6,47,48].

## 5. Conclusions

To elevate reporting standards in physical and rehabilitation medicine, critical methodological and reporting enhancements must address persistent gaps in intervention transparency, trial design, and reproducibility. Besides improving the reporting gaps outlined in the present study, mandatory adoption of specialized reporting guidelines would be of added value, particularly the TIDieR-Rehab checklist, which extends the original TIDieR framework to rehabilitation-specific elements like dosage parameters, customization, and adverse effects documentation [49]. This ensures detailing of intervention components like provider qualifications, delivery setting, and fidelity monitoring, directly addressing common deficiencies in describing “who, how, and where” interventions occur. Strengthened methodological rigor requires adherence to extensions of the CONSORT statement tailored for nonpharmacologic trials (CONSORT-NPT), which provide structured guidance for complex rehabilitation interventions including sample sizing, blinding protocols and randomization integrity, which were predominantly not reported from our examined articles [50].

Future studies might also look into how much journals encourage adequate reporting in this field, as their involvement could be the key driver of the implementation of good practices. Prospective registration of rehabilitation trials with detailed protocols including main outcomes would mitigate retrospective design limitations like subjective interpretation bias while standardizing data collection [51]. These changes collectively address the field’s reproducibility crisis, ensuring interventions are replicable, clinically applicable, and methodologically precise.

The implications of our study can generally inform the way future studies on psychological interventions for scoliosis are reported. Besides improving basic reporting, studies should focus on testing their applicability and measuring adoption barriers in diverse settings. Also, prospective studies that give authors instructions for better writing using the TIDieR checklist, then compare results with a control group of studies that did not have the same instructions, would be useful in assessing the impact reporting guidelines would have in improving the generation and quality of research in this field. Ultimately, a commitment to rigorous reporting and methodological innovation will not only enhance the scientific quality of individual studies but also accelerate the translation of research findings into clinical practice. This will ensure that patients with scoliosis benefit from evidence-based psychological interventions that are both effective and accessible.

## Figures and Tables

**Table 1:** Completeness of reporting psychological interventions for scoliosis according to TIDieR checklist.

TIDieR Item	TIDieR Analysis of Published Articles (N = 18)		
	Yes	No	Unclear
Item 1—Brief name	18	0	0
Item 2—Why	18	0	0
Item 3—What materials	10	2	6
Item 4—What methods	14	1	3
Item 5—Who provided	6	5	7
Item 6—How	8	6	4
Item 7—Where	3	10	5
Item 8—When and how much	11	2	5
Item 9—Tailoring	3	1	14 non-applicable
Item 10—Modifications	1	0	17 non-applicable
Item 11—How well-planned	1	1	16 non-applicable
Item 12— How well-actual	1	1	16 non-applicable

TIDieR—Template for Intervention Description and Replication. We use the rating of “Non-applicable” when the relevant item is not planned within the intervention methods. We use the rating of “Unclear” when the relevant item is reported with insufficient precision and details.

**Table 2:** Completeness of reporting of randomized controlled trials of psychological intervention for scoliosis according to CONSORT checklist.

CONSORT Item	*n/N*
1a. Identification as a randomized trial in the title	3/8
1b. Structured summary of trial design, methods, results, and conclusions	5/8
3a. Description of trial design (such as parallel, factorial) including allocation ratio	1/8
4b. Settings and locations where the data were collected	5/8
7a. How sample size was determined	3/8
8b. Type of randomization; details of any restriction (such as blocking and block size)	1/8
9. Mechanism used to implement the random allocation sequence	2/8
10. Who generated the random allocation sequence, who enrolled participants, and who assigned participants to interventions	1/8
11a. If done, who was blinded after assignment to interventions (for example, participants, care providers, those assessing outcomes) and how	1/8
13b. For each group, losses and exclusions after randomization, together with reasons	5/8
14a. Dates defining the periods of recruitment and follow-up	3/8
15. A table showing baseline demographic and clinical characteristics for each group	4/8
16. For each group, number of participants (denominator) included in each analysis and whether the analysis was by original assigned groups	3/8
21. Generalizability (external validity, applicability) of the trial findings	2/8
23. Registration number and name of trial registry	1/8
24. Where the full trial protocol can be accessed, if available	1/8
25. Sources of funding and other support (such as supply of drugs), role of funders	5/8

Other CONSORT items not mentioned in Table 2 were adequately reported (see Supplementary Material S3).

**Table 3:** Completeness of reporting of observational studies of psychological intervention for scoliosis according to STROBE checklist.

STROBE Item	*n/N*
4. Present key elements of study design early in the paper	3/5
5. Describe the setting, locations, and relevant dates, including periods of recruitment, exposure, follow-up, and data collection	3/5
6a. Give eligibility criteria, sources and methods of selection, and follow-up methods	3/5
8. For each variable of interest, give sources of data and details of methods of assessment (measurement). Describe comparability of assessment methods if there is more than one group	1/5
9. Describe any efforts to address potential sources of bias	2/5
10. Explain how the study size was arrived at	1/5
12c. Explain how missing data were addressed	0/5
12d. If applicable, explain how loss to follow-up was addressed	2/5
13a. Report numbers of individuals at each stage of study—e.g., numbers potentially eligible, examined for eligibility, confirmed eligible, included in the study, completing follow-up, and analyzed	1/5
13b. Give reasons for non-participation at each stage	2/5
13c. Consider use of a flow diagram	1/5
14b. Indicate number of participants with missing data for each variable of interest	0/5
16a. Give unadjusted estimates and, if applicable, confounder-adjusted estimates and their precision (e.g., 95% confidence interval). Make clear which confounders were adjusted for and why they were included	2/5
16b. Report category boundaries when continuous variables were categorized	0/5
21. Discuss the generalizability (external validity) of the study results	3/5
22. Give the source of funding and the role of the funders for the present study and, if applicable, for the original study on which the present article is based	3/5

Other STROBE items not mentioned in Table 3 were adequately reported (see Supplementary Material S3).

**Table 4:** Completeness of reporting of nonrandomized evaluations of psychological intervention for scoliosis according to TREND checklist.

TREND Items	*n/N*
3. Participants	0/5 (all partial)
7. Sample size	0/5
9. Blinding	0/5
12. Participant flow	0/5 (all partial)
13. Recruitment	3/5
14. Baseline data	0/5 (all partial)

Other TREND items not mentioned in Table 4 were adequately reported (see Supplementary Material S3).

## Data Availability

Supporting Data is available in the Supplementary Materials.

## Supplementary Materials

The following supporting information can be downloaded at https://www.mdpi.com/article/10.3390/healthcare13222872/s1, Supplementary Material S1: Criteria for assessment, Supplementary Material S2, Supplementary Material S3.

## Abbreviations

The following abbreviations are used in this manuscript:

AIS	Adolescent idiopathic scoliosis
EQUATOR	Enhancing the QUAlity and Transparency Of health Research
TIDieR	Template for Intervention Description and Replication
CONSORT	Consolidated Standards Of Reporting Trials
STROBE	STrengthening the Reporting of OBservational studies in Epidemiology
TREND	Transparent Reporting of Evaluations with Nonrandomized Designs
