# Supplementary material for: Completeness of Reporting and Intervention Description in Articles on Psychological Interventions for Pediatric Patients with Adolescent Idiopathic Scoliosis: A Meta-Research Study
Source: Healthcare (Basel). 2025 Nov 12;13(22):2872. doi: 10.3390/healthcare13222872 (PMC12652975; doi:10.3390/healthcare13222872)
Supplement: Supplementary file 1 [file healthcare-13-02872-s001.zip › Supplement S1.pdf]

Criteria for assessment

Item 1: Yes, if the name of the paper is clearly stated and from the title it can be understood what it is about.
No, if the topic of the paper is completely unclear from the title.
Unclear, if the topic of the paper is mentioned in the title, but cannot be fully understood
Item 2: Yes, if the purpose of the paper is clearly stated in the introduction.
No, if the goal and purpose of the paper is not identified in the introduction.
Unclear, if the reason for writing the paper can be assumed, but is not clearly stated.
Item 3: Yes, if the materials are described in sufficient detail and where they can be accessed.
No, if the materials are not mentioned or explained in the methodology.
Unclear, if the materials are listed, however, they lack detail in the description or authors did not specify where they can be accessed.
Item 4: Yes, if the procedures used are clearly and fully stated, and explained in detail.
No, if the procedures used are not specified or are very vague and poorly explained.
Unclear, if the procedures used are stated and explained, but lacking a detailed description.
Item 5: Yes, if it is stated for each part of the intervention who carried it out, what is his profession, and how much experience he has in that field.
No, if it is not specified at all for the intervention who conducted it, what is his profession, and how much experience he has in that field.
Unclear, if it is stated for some parts of the intervention who carried it out, what is his profession, and how much experience he has in that field, and for some parts there is no adequate data.
Item 6: Yes, if it is clearly stated how the intervention was delivered, by what means and whether it was individually or in a group.
No, if it is not stated at all how the intervention was delivered, by what means and whether it was individually or in a group.
Unclear, if it is partially stated how the intervention was delivered, by what means and whether it was individually or in a group, with the lack of necessary details.
Item 7: Yes, if it is fully specified where the intervention took place, including state, city, hospital and department.
No, if it is not specified where the intervention took place.
Unclear, if partially stated where the intervention took place, incompletely reported state, city, hospital and department.
Item 8: Yes, if the time when the intervention was carried out, and how many times and in which dose/intensity the subjects received the intervention procedure are described in detail.
No, if the time when the intervention was carried out, and how many times and in which dose/intensity the subjects received the intervention procedure were not mentioned or explained.
Unclear, if the time when the intervention was carried out, and how many times and in which dose/intensity the subjects received the intervention procedure are not explained in sufficient detail.
Item 9: Yes, if the intervention was planned to be adapted, individualized, and the process is described completely.
No, if the intervention was planned to be adapted, individualized, and the process is not described.
Unclear, if the intervention was planned to be adapted, individualized, and the process is described partially or is lacking significant details.

Non-applicable, if the intervention wasn’t planned to be adapted or individualized.
Item 10: Yes, if the intervention was modified and changes were explained in detail.
No, if the intervention was modified and changes were not explained at all.
Unclear, if the intervention was modified and changes were partially explained, lacking in detail.
Non-applicable, if the intervention was not modified during the course of the study.
Item 11: Yes, if intervention adherence or fidelity was assessed and described in full detail.
No, if intervention adherence or fidelity was assessed, but was not described.
Unclear, if intervention adherence or fidelity was assessed and described partially.
Non-applicable, if intervention adherence or fidelity was not assessed.
Item 12: Yes, if intervention adherence or fidelity was assessed, described actual results of that assessment in detail.
No, if intervention adherence or fidelity was assessed, failed to describe actual results of that assessment.
Unclear, if intervention adherence or fidelity was assessed, partially described actual results of that assessment.
Non-applicable, if intervention adherence or fidelity was not assessed.
